# Optimization of Protease Treatment Conditions for *Chlorella pyrenoidosa* Protein Extraction and Investigation of Its Potential as an Alternative Protein Source

**DOI:** 10.3390/foods13030366

**Published:** 2024-01-23

**Authors:** Kyung-Jin Cho, Min-Ung Kim, Geum-Jae Jeong, Fazlurrahman Khan, Du-Min Jo, Young-Mog Kim

**Affiliations:** 1 Department of Food Science and Technology, Pukyong National University, Busan 48513, Republic of Korea; rudwls803342@gmail.com (K.-J.C.);; 2 Marine Integrated Biomedical Technology Center, The National Key Research Institutes in Universities, Pukyong National University, Busan 48513, Republic of Korea; 3 Research Center for Marine Integrated Bionics Technology, Pukyong National University, Busan 48513, Republic of Korea; 4 Institute of Fisheries Sciences, Pukyong National University, Busan 48513, Republic of Korea

**Keywords:** *Chlorella pyrenoidosa*, enzymatic hydrolysis, optimization, antioxidant, protein source

## Abstract

This study aimed to determine enzymes that effectively extract *Chlorella pyrenoidosa* proteins and optimize the processing conditions using response surface methods. Furthermore, the potential of enzymatically hydrolyzed *C. pyrenoidosa* protein extract (CPE) as a substitute protein source was investigated. The enzymatic hydrolysis conditions for protein extraction were optimized using single-factor analysis and a response surface methodology–Box–Behnken design. The R^2^ value of the optimized model was 0.9270, indicating the reliability of the model, and the optimal conditions were as follows: a hydrolysis temperature of 45.56 °C, pH 9.1, and a hydrolysis time of 49.85 min. The amino acid composition of CPE was compared to that of *C. pyrenoidosa* powder (CP), which was found to have a higher content of essential amino acids (EAA). The electrophoretic profiles of CP and CPE confirmed that CPE has a low molecular weight. Furthermore, CPE showed higher antioxidant activity and phenol content than CP, with ABTS and DPPH radical scavenging abilities of 69.40 ± 1.61% and 19.27 ± 3.16%, respectively. CPE had high EAA content, antioxidant activity, and phenol content, indicating its potential as an alternative protein source. Overall, in this study, we developed an innovative, ecofriendly, and gentle enzymatic hydrolysis strategy for the extraction and refinement of Chlorella proteins.

## 1. Introduction

The world’s population is increasing at an alarming pace each year, posing a major problem in sustaining a sufficient food supply to satisfy the increasing demand [1]. Owing to greenhouse gas emissions, environmental pollution, ecosystem destruction, and the overexploitation of land and resources, a new food production approach is needed. Protein is a macronutrient that will be in limited supply in the future, necessitating the development of alternative protein sources and more efficient means of production than the current production technologies. 

Microalgae are single-celled microorganisms that thrive in freshwater and oceans, making them suitable for large-scale cultivation. They are currently regarded as sustainable raw resources for biofuel production [2]. In addition, microalgae are rich in nutrients, such as lipids, carbohydrates, and proteins, and have been utilized as raw materials for food and as a source of micronutrients, such as vitamins, pigments, and minerals [3]. Microalgae are particularly attracting attention for use in functional foods, food additives, and supplements within the food industry, as they have been shown to possess potential for application in the field of nutrition [4]. The bioenergy, feed, and food industries extensively use Chlorella, *Chlorella* spp., which is one of the most important microalgae. The protein content of Chlorella is much higher than that of other plant substances, ranging from 50 to 60% per dry weight [5]. In addition, Chlorella proteins exhibit physiological activities, such as immunity boosting, antioxidant, and anti-inflammatory properties, and have excellent nutritive qualities because of the presence of both essential and non-essential amino acids. The cell wall structure is composed of an inner cell wall layer of cellulose polymer and an outer cell wall layer of algae, making it very rigid [1]. Consequently, it is of utmost importance to break down the cell wall and remove the internal proteins. Ultrasonication [6], bead milling [7], microwave radiation [8], and homogenizers [9] are all examples of physical approaches that have been used for breaking down the cell wall, and bioactive molecules, such as proteins, are efficiently eluted. However, these methods require tremendous energy and provide excessive heat to the extract, which may result in denaturation of the proteins and damage to the bioactive molecules [10,11,12]. However, enzymatic hydrolysis for protein extraction from Chlorella has high selectivity and does not require energy, indicating that it may be regarded as a novel processing approach [13]. Currently, research on protein extraction using enzymes is underway, and it has been observed that the yield of proteins extracted from Chlorella varies depending on the type of enzyme used. Furthermore, studies are being performed to improve the protein extraction yield through treatment with two or more enzymes in succession or by combining physical approaches with enzyme treatment [14,15]. Regardless of the high extraction yield, these approaches are likely to be challenging for implementation in the food industry because of the increased processing time and costs. In particular, there is a considerable lack of research on protein extraction from Chlorella and protein materialization for use in the food industry. Therefore, the purpose of this study was to identify the enzymes capable of efficiently extracting proteins from Chlorella. In addition, response surface methodology (RSM) was used to optimize the treatment conditions for the chosen enzymes. Here, we report a novel, eco-friendly, and gentle enzymatic hydrolysis approach for the extraction of proteins from Chlorella.

## 2. Materials and Methods

### 2.1. Materials and Chemicals

*C. pyrenoidosa* powder (CP) used in the experiment was purchased from Jeongwoodang Co., Ltd. (Seoul, Republic of Korea). Microbial protease from *Bacillus* spp. (EC 3.4.21.14), papain from *Carica papaya* fruit (EC 3.4.22.2), bromelain from pineapple stem (EC 3.4.22.32), cellulase from *Trichoderma reesei* (EC 3.2.1.4), and viscozyme L from *Aspergillus* sp. (cellulolytic enzyme mixture) were purchased from Sigma-Aldrich (St. Louis, MO, USA) (Table 1). All other chemicals and reagents were of analytical grade and are listed as follows: sodium dodecyl sulfate, acrylamide, Coomassie brilliant blue, methanol, 1,2-bis (dimethylamino) ethane, 2,2-azino-bis (3-ethylbenzothiazoline-6-sulfonic acid) diammonium salt (ABTS), potassium persulfate, 2,2-diphenyl-1-picrylhydrazyl (DPPH), gallic acid, Folin–Ciocalteu’s phenol reagent, and sodium carbonate were acquired from Sigma-Aldrich. Trichloroacetic acid (TCA) was purchased from Samchun Chemical Co. (Pyeongtaek, Republic of Korea).

### 2.2. Enzymatic Hydrolysis of C. pyrenoidosa

Microbial proteases, papain, bromelain, cellulase, and viscozyme L were used to extract proteins from *C. pyrenoidosa* via enzymatic hydrolysis; their characteristics are presented in Table 1. First, 10 g of CP was mixed with 200 mL of distilled water to make a 5% (*w*/*v*) suspension, and then, 3% of the enzyme (*w*/*v*) was added compared to the substrate. The CP suspension without enzyme treatment was set as the control group. The optimum hydrolysis reaction pH for each enzyme was adjusted using 1 N NaOH and HCl, and enzymatic hydrolysis was performed in a shaking incubator for 0, 3, 6, 9, 12, 15, and 18 h at the optimal hydrolysis temperature. The enzyme was inactivated by heat treatment (100 °C, 10 min) after hydrolysis, and the supernatant was separated by centrifugation (10,000× *g*, 20 min; Hanil Scientific, Inc., Gimpo, Republic of Korea) and used for the experiment.

### 2.3. Determination of Protein Extraction Yield

Protein concentration was measured using a bicinchoninic acid assay (BCA; Pierce™ BCA Protein Assay Kit, Thermo Fisher Scientific, Rockford, IL, USA) according to a previously described method [21]. In 96-well plates, 200 µL of dye solution was mixed with 25 µL of sample. The mixture was incubated at 37 °C for 30 min. Bovine serum albumin was used as a standard material to construct a standard curve for protein quantification. The protein yield extracted from *C. pyrenoidosa* was calculated using the following equation:

Protein extraction yield (%) = (P_cpe_ − P_e_)/P_cp_ × 100%

where P_cp_ is the protein content of CP (mg/mL), P_cpe_ is the protein content of the enzyme-treated *C. pyrenoidosa* protein extract (CPE, mg/mL), and P_e_ is the protein content of the enzyme.

### 2.4. Experimental Design and Optimization for Enzymatic Hydrolysis Conditions

The software Design Expert version 7.0 (Stat-Ease Inc., Minneapolis, MN, USA) was used in this study for constructing the experimental design and predicting the optimum enzymatic hydrolysis conditions for protein extraction from *C. pyrenoidosa*, data analysis, and building models. An RSM–Box–Behnken design (BBD) with three variables was used to demonstrate the response patterns and create models. The three variables employed in this study were hydrolysis temperature (A, °C), initial pH (B), and hydrolysis time (C, min), with each variable having three values (−1, 0, +1). The yield of protein extracted from *C. pyrenoidosa* (%) was chosen as the dependent variable Y. To ensure method repeatability, the experimental design included 17 experimental points and five central point repeats. Following these experiments, a statistical analysis was performed, and an optimization model based on a second-order polynomial was developed. The estimated values of the dependent variables were validated by comparing them with the values obtained from actual experiments under statistically calculated optimal conditions.

### 2.5. Characteristic Analysis of CP and CPE

The quality characteristics of CPE under optimal conditions and CP before enzymatic hydrolysis were compared. The freeze-dried supernatant was used as the sample.

#### 2.5.1. Amino Acid Composition

The total and free amino acid contents of CP and CPE were determined as previously described [22]. The sample used for the analysis of total amino acids was hydrolyzed at 110 °C for 24 h by adding 10 mL of 6 N HCl. Afterward, the sample was dried at 40 °C by an evaporator (Hei VAP Expert; Heidolph, Schwabach, Germany) to remove the traces of HCl. The sample was then diluted with a 0.02 N HCl solution and used as a test solution. For free amino acids, samples were mixed with equal amounts of 16% TCA solution and shaken for 15 min. Afterward, they were then centrifuged (3000× *g*, 15 min), and the supernatant was used as the test solution. The total and free amino acids were analyzed using an automatic analyzer (L-8900; Hitachi High-Technologies Corp., Tokyo, Japan). The column used was a Hitachi HPLC Packed column (ion-exchange resin, 4.6 mm i.d., 60 mm length, 3 µm particle size; Tokyo, Japan), and a visible detector (Hitachi High-Technologies Corp.) was set to measure the optical densities at 440 and 570 nm.

#### 2.5.2. Sodium Dodecyl Sulfate–Polyacrylamide Gel Electrophoresis (SDS–PAGE) Analysis

The protein fractions of CP and CPE were determined by SDS–PAGE using a Mini-Protein BioRad electrophoresis system (BioRad Laboratories Ltd., Watford, UK). Electrophoresis was carried out using 12% separating gel and 5% stacking gel as per a previously described procedure with some modifications [23]. The samples were mixed with 5X SDS–PAGE sample buffer at a ratio of 4:1 (*v*/*v*) and heated at 100 °C for 10 min. After loading 20 µL of samples into the well, the electrophoresis system was run for 2 h at 110 V. The gels were stained with Coomassie Blue G-250 and destained using an acetic acid/methanol/water (1:4:5, *v*/*v*/*v*) solution. HiQ BluePlus Protein Markers (A20028; BioD, Gwangmyeong, Republic of Korea) were used in the mass range of 10–245 kDa to identify the fractions.

#### 2.5.3. Antioxidant Properties

The DPPH radical scavenging activity was determined as per a previously described procedure [24]. Each sample (0.5 mL) was combined with 1 mL of 0.15 mM DPPH solution and incubated at 25 °C for 30 min before being measured at 517 nm using a microplate reader (BioTek, Winooski, VT, USA). The DPPH radical scavenging activity was calculated using the following equation:

DPPH scavenging activity (%) = (Abs_control_ − Abs_sample_)/Abs_control_ × 100%

The ABTS radical scavenging activity was examined using a previously described method with some modifications [25]. The ABTS radical solution was prepared at 7 mM, including 2.4 mM potassium persulfate, and incubated for 16 h in a dark room. The ABTS radical solution was diluted to an absorbance of 0.700 ± 0.005 at 734 nm. Then, 500 µL of each sample was reacted with 500 µL of ABTS radical solution in the dark at 25 °C for 6 min, and the absorbance was measured at 734 nm using a microplate reader (BioTek). The ABTS radical scavenging activity was calculated using the following equation:

ABTS scavenging activity (%) = (Abs_control_ − Abs_sample_)/Abs_control_ × 100%

#### 2.5.4. Total Phenolic Content (TPC)

The TPC was determined using previously described procedures with some modifications [26]. To 200 µL of the sample, 1 mL of 1 N Folin–Ciocalteu’s phenol reagent was added and reacted at room temperature (20 ± 5 °C) for 3 min. Subsequently, 2 mL of 10% sodium carbonate was added and reacted in the dark at room temperature for 30 min. The reaction mixture was centrifuged (15,928× *g* for 5 min at 4 °C), 200 µL of the supernatant was dispensed onto a 96-well plate, and the absorbance at 760 nm was measured using a microplate reader. Gallic acid was used as the standard, and its concentration was diluted two-fold to create a standard curve. The results are expressed as gallic acid equivalents (µM GAE/mL).

### 2.6. Statistical Analysis

All experiments were conducted in triplicate. The results were analyzed using SPSS 27 (IBM SPSS Advanced Statistics, Chicago, IL, USA) to calculate the mean and standard deviation for each test group. Duncan’s multiple range analysis was used to determine statistically significant differences between the data. The significance level was set at 5% (*p* < 0.05).

## 3. Results

### 3.1. Selection of Enzyme for Optimization

The cell walls of Chlorella are composed of polysaccharides and glycoproteins [27]. Therefore, in this study, we investigated the effects of proteases (microbial protease, papain, and bromelain) and cellulases (cellulase and viscozyme L) on the yield of Chlorella protein extraction. Table 2 shows the yield of protein extraction from *C. pyrenoidosa* based on the enzyme type and reaction time used to choose an enzyme that may be employed for protein extraction from *C. pyrenoidosa*. The differences in the yield of protein extraction were significant and were in the following order: microbial protease, papain, bromelain, viscozyme L, and cellulase (*p* < 0.05). After 3 h of microbial protease treatment, the protein extraction yield was 34.54 ± 2.48%, and it rapidly declined. After 18 h of treatment, the protein extraction yields for papain and bromelain were 18.22 ± 0.47% and 10.02 ± 0.32%, respectively. Endopeptidases such as microbial proteases, papain, and bromelain are effective in decomposing the cell walls of plant materials such as Chlorella [28]. Cunha et al. found that effective protein extraction was achievable when proteases, such as alkalase, were used, and a similar trend was observed in our study [29]. Although papain and bromelain are endopeptidases, their protein extraction yield is lower than that of microbial proteases. According to Lee et al., microbial proteases use 1 N NaOH to achieve a pH of 10, which is considered to play a role in the breakdown of the cell wall [30]. After 6 h of treatment, the concentrations of cellulase and viscozyme L were 6.57 ± 0.27% and 9.23 ± 0.70%, respectively. Notably, the yield of protein extraction was lower than that of the proteases. Therefore, the microbial protease with the highest protein extraction yield from *C. pyrenoidosa* was chosen to optimize the enzymatic hydrolysis conditions for effective *C. pyrenoidosa* protein extraction.

### 3.2. Results of Single-Factor Analysis and Experimental Design

**Figure 1:**
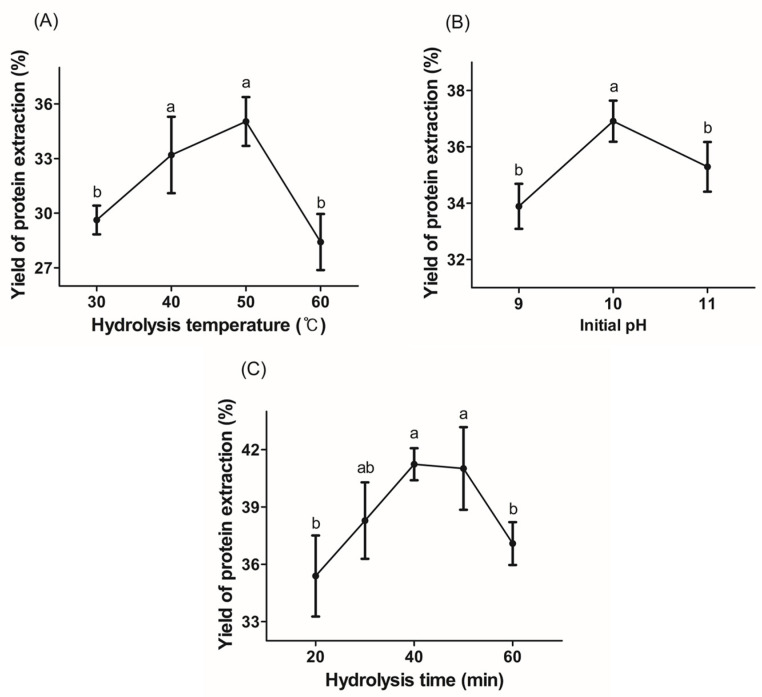
Protein extraction yield from *Chlorella pyrenoidosa* by microbial protease under hydrolysis conditions: (**A**) hydrolysis temperature (°C), (**B**) initial pH, and (**C**) hydrolysis time (min). Values with different letters vary significantly (*p* < 0.05).

Before employing RSM–BBD to optimize the microbial protease treatment parameters, a single-factor analysis was performed to determine the range of effects of the three independent variables (hydrolysis temperature, A; initial pH, B; and hydrolysis time, C). Figure 1 shows the effects of one factor during microbial protease treatment on the yield of proteins extracted from *C. pyrenoidosa*, which is the dependent variable. All three variables had a positive effect on protein extraction yield. Protein extraction yield was considerably higher at 40 and 50 °C for the microbial protease reaction temperature (Figure 1A), at an initial pH of 10 (Figure 1B), and at 40 and 50 min of reaction time (Figure 1C). Temperature, pH, and time during the enzymatic process affect the yield of Chlorella protein extraction, which is a dependent variable [31]. As a result, for the optimization of microbial protease treatment conditions, the range of independent variables was chosen to be 30–50 min for hydrolysis time (A), 9–11 for initial pH (B), and 40–60 °C for hydrolysis temperature (C).

### 3.3. Optimization of the Enzymatic Hydrolysis Conditions

The effect between the factors can be validated by comparing each coefficient, making the quadratic equation helpful in the optimization analysis [32,33]. In addition, it was used to predict the optimization model by employing variable factors based on a variance analysis. Consequently, an RSM–BBD experiment was developed with a number of different variables to build a numerical model for producing equation coefficients capable of efficiently extracting *C. pyrenoidosa* proteins using microbial proteases. The actual values obtained from the microbial protease treatment experiments are presented in Table 3 and are arranged according to the experimental level and variable range of the single factor examined earlier. The predicted values computed using the quadratic equation included a list of anticipated values corresponding to the 17 input points for the microbial protease treatment conditions. The yield of *C. pyrenoidosa* protein extraction was highest at 39.39% in No. 14 (A: 50 °C, B: pH 9, and C: 50 min) and lowest at 31.74% in No. 2 (A: 40 °C, B: pH 11, and C: 40 min). The experimental results were fitted using the following second-order polynomial equations:

Y = −99.86311 + 3.27244 A + 8.71967 B + 0.57823 C + 0.027155 AB − 0.010520 AC − 0.073515 BC − 0.030525 A^2^ − 0.39049 B^2^ + 0.00975255 C^2^

where Y denotes the protein extraction yield from *C. pyrenoidosa* and A, B, and C denote the hydrolysis time, initial pH, and hydrolysis temperature, respectively.

**Figure 2:**
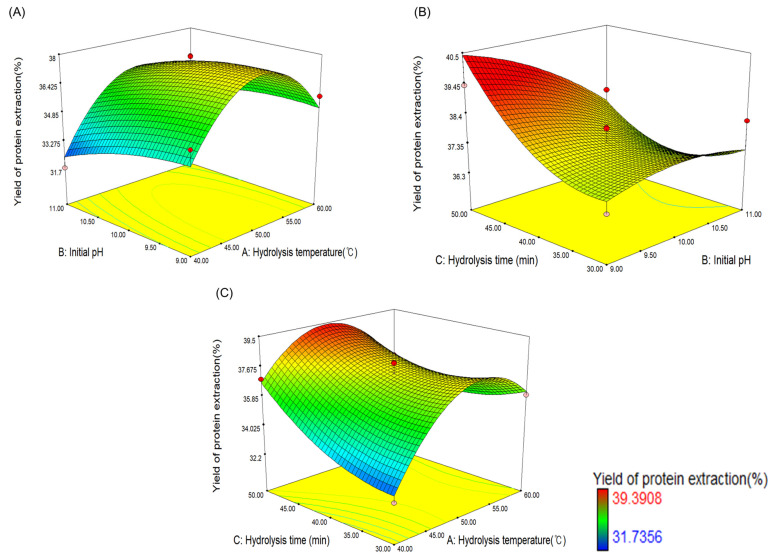
Three-dimensional analysis of the effects of (**A**) hydrolysis temperature (°C) and initial pH, (**B**) hydrolysis temperature (°C) and hydrolysis time (min), and (**C**) hydrolysis time (min) and initial pH on protein extraction yield from *Chlorella pyrenoidosa* powder. The Color indicates protein extraction yield. Red indicates high protein extraction yield, and blue indicates low.

Analysis of variance (ANOVA) was used to assess the statistical significance of the derived quadratic models (Table 4). In Table 4, the findings of this study are shown as the total square, mean square, *F*-value, *p*-value of the model, the variables and their interactions, lack of fit, and others. In general, if the *F*-value is greater than 1, the optimization model is valid [34], and the optimization model in this study was determined to be valid, with an *F*-value of 7.58. The lack of fit was used to evaluate the suitability of the model because it provides information about the suitability of the model by checking for errors caused by model noise [35]. The lack of fit of the model was 0.1064, which is greater than 0.05, indicating that the optimized model was significant. The *p*-value describes the significance of each variable and the strength of the interaction between the variables. As shown in Table 4, the independent variables of microbial protease treatment conditions, namely hydrolysis temperature (A, °C), initial pH (B, pH), and hydrolysis time (C, min), were found to be statistically significant (*p* < 0.05). The quadratic term A^2^ was statistically significant (*p* < 0.05), but B^2^ and C^2^ were not found to be significant. Moreover, in the interaction of each variable, only AC (hydrolysis temperature and time) was statistically significant, whereas AB and BC were not. The equation is displayed as a 3D graph to confirm the interaction effects of each factor in Figure 2. Figure 2B shows a slight curve, indicating that AC was statistically significant, but the effect was minimal.

Table 5 presents the results of the regression analyses. The R^2^ value for the *C. pyrenoidosa* protein extraction model was 0.9270, which was greater than 0.9. The closer the R^2^ value is to 1, the greater the correlation between the actual and predicted values, suggesting that the developed model is reliable, as previously reported [36]. It was determined that the model is acceptable because the adequacy precision (AP), computed by comparing the ranges of the anticipated values at the designed points with the average prediction error, is greater than four [37]. According to the findings of this investigation, the AP value was 10.749, indicating that the optimization model is acceptable. Thus, the optimized model in this study was statistically significant.

### 3.4. Validation of the Optimized Microbial Protease Treatment Condition

Single-factor analysis and RSM–BBD methods were used to achieve the optimal enzymatic treatment conditions for extracting *C. pyrenoidosa* protein. Based on the RSM–BBD results, the optimal conditions for attaining the highest possible yield of *C. pyrenoidosa* protein extraction are listed in Table S1. An enzymatic hydrolysis temperature of 45.56 °C, initial pH of 9.1, and enzymatic hydrolysis time of 49.85 min were the conditions that were found to be optimal. A verification test was performed using the closest integer to validate the developed optimization parameters. The experiment was carried out using the following parameters: the temperature of the enzymatic hydrolysis was 45.6 °C, the initial pH was 9.1, and the enzymatic hydrolysis time was 49.85 min. Consequently, the difference between the actual and predicted values was statistically significant (*p* < 0.05), indicating that the optimization model that made use of the RSM was statistically valid.

### 3.5. Amino Acids Analysis of CPE

In the process of determining the nutritional content, digestibility, and function of a product, it is widely acknowledged that the amino acid composition of the protein extracted from Chlorella, *C. pyrenoidosa*, is of critical importance [38]. Therefore, to evaluate the quality of the amino acids in CPE optimized in this study, the total and free amino acids were compared with CP; the results are shown in Table 6 and Table 7. The total amino acids were 17, and the contents of total amino acids were 43,034.8 ± 69.8 and 56,396.3 ± 517.7 mg/100 g for CP and CPE, respectively, with an increase in the levels of all amino acids, except lysine (Table 6). Moreover, the content of total essential amino acids of CPE was 22,849.1 ± 194.9 mg/100 g, which was higher than that of CP (18,841.3 ± 129.2 mg/100 g). High-quality proteins require essential amino acids [39]. Thus, this supports the idea that the protein quality of CPE is better than that of CP because CPE contains a greater quantity of essential amino acids than CP. Furthermore, the content of branched-chain amino acids (BCAAs; valine, isoleucine, and leucine), which play roles in muscle development and control of the central nervous system [40], was higher in CPE (10,941.3 mg/100 g) than in CP (8745.3 mg/100 g). Thus, the BCAA content increased in CPE.

The free amino acid contents of CP and CPE were 896.0 ± 24.7 and 3041.2 ± 74.7 mg/100 g, respectively (Table 7). As for the total amino acids, the essential free amino acid content of CPE was 745.5 ± 23.1 mg/100 g, which was 3.5 times higher than that of CP (213.0 ± 4.4 mg/100 g). In particular, hydrophobic and negatively charged amino acids, such as alanine, glutamic acid, and proline, are examples of amino acids that possess bioactive properties such as antioxidant activity [41,42]. It is anticipated that these amino acids abundantly existing in CPE will result in an increase in its bioactive properties, such as antioxidant activity. In addition, the content of glutamic acid enhancing the umami flavor of food was 541.4 ± 10.6 mg/100 g, the second highest among free amino acids in CPE [43]. β-amino isobutyric acid (BAIBA), which is beneficial for muscle mass development and protein synthesis, was not detected in CP, but had the highest content among free amino acids in CPE at 650.8 ± 23.6 mg/100 g [44]. The content of γ-amino-n-butyric acid (GABA), which improves cerebral blood flow, neuronal relaxation, and memory, was also higher in CPE (117.2 ± 5.5 mg/100 g) than that of CP (9.9 ± 0.3 mg/100 g) [45]. Therefore, based on the analysis of total and free amino acids, CPE was found to possess a higher content of essential amino acids than CP. Furthermore, it contains many functional and flavor substances, which makes it advantageous for use in food as an alternative protein material.

### 3.6. Protein Profiles of CPE

**Figure 3:**
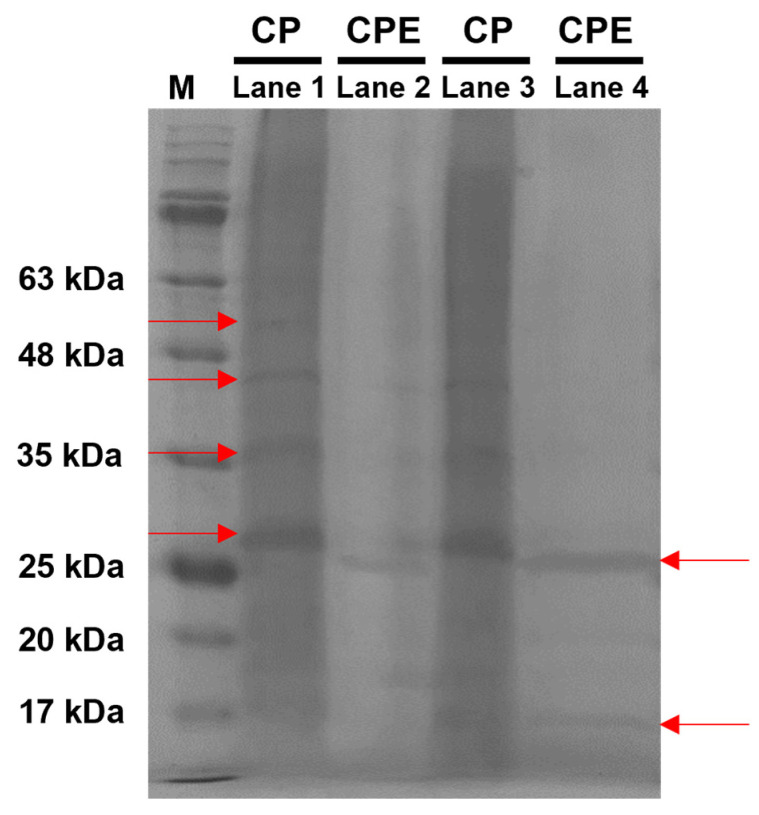
SDS-PAGE analysis of protein fractions from *Chlorella pyrenoidosa* powder (CP) and *C. pyrenoidosa* protein extracted by microbial protease (CPE). M denotes the protein molecular mass marker (10–245 kDa). Lanes 1 and 3 are for CP, and lanes 2 and 4 for CPE.

The protein profiles of CP and CPE determined using electrophoresis are shown in Figure 3. In the case of CP, bands were observed at approximately 52, 45, 35, and 27 kDa, whereas in the case of CPE, bands were observed at 25 and slightly below 17 kDa. Sharma et al. reported that the molecular weight of proteins varies depending on the culture conditions of Chlorella [46]. In addition, the untreated *C. pyrenoidosa* protein sizes of 23, 26, and 35 kDa were comparable to the protein size of CP. These values were consistent across all culture conditions. The microbial protease is responsible for converting the Chlorella protein into smaller molecules; thus, the CPE protein is possibly smaller than the CP protein. Enzymatic hydrolysis is an essential mechanism affecting the physicochemical and nutritional qualities of food [47]. This process involves the breakdown of proteins into large- and small-molecule peptides, as well as free amino acids. As a result, the CPE optimized in this study is a low-molecular-weight protein that is simple to digest and absorb. It has the potential to be used as an alternative to protein materials in the production of protein-rich foods.

### 3.7. Antioxidant Activity and Phenolic Content of CPE

**Figure 4:**
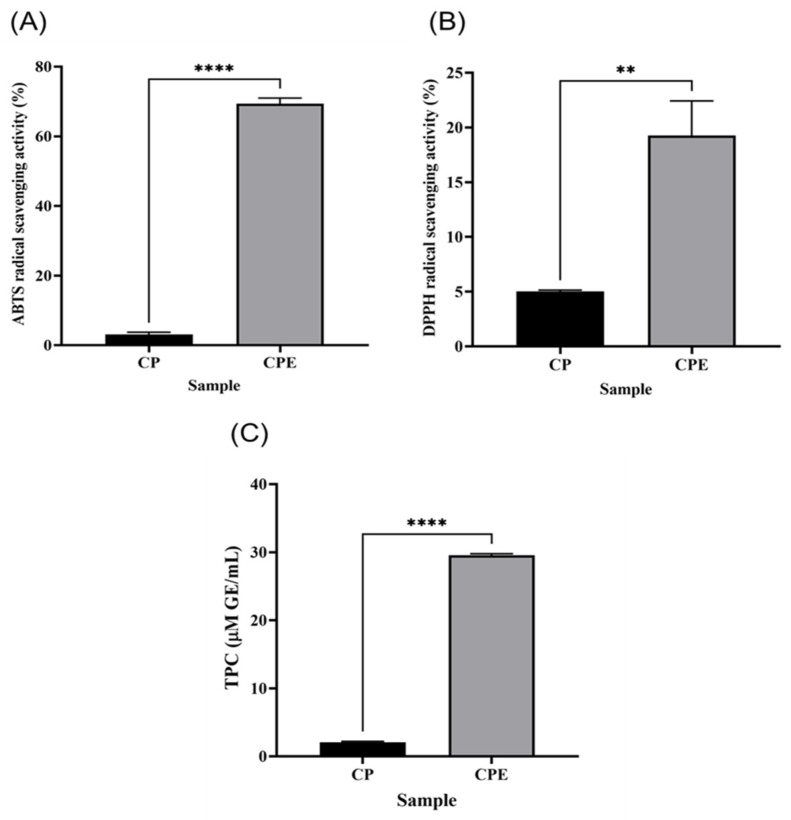
Antioxidant capacity and phenolic content of *Chlorella pyrenoidosa* powder (CP) and *C. pyrenoidosa* protein extracted by microbial protease (CPE). (**A**) ABTS assay, (**B**) DPPH assay, and (**C**) total polyphenol (TPC) content. Each value is mean ± SD (*n* = 3). ** *p* < 0.01, **** *p* < 0.0001 based on an independent *t*-test comparing two samples (CP and CPE).

The ABTS and DPPH radical scavenging abilities and TPC results for CP and CPE are shown in Figure 4. ABTS radical scavenging activity was determined based on the principle of reducing ABTS cation radicals generated by potassium peroxidase [48]. The ABTS radical scavenging abilities of CP and CPE were 3.10 ± 0.65% and 69.40 ± 1.61%, respectively (*p* < 0.0001; Figure 4A). The DPPH radical scavenging ability of CPE was 19.27 ± 3.16%, which was significantly higher than that of CP (5.01 ± 0.13%, *p* < 0.01; Figure 4B). The antioxidant activity of protein hydrolysates is especially influenced by amino acid compositions in hydrolysates [48]. Shi et al. also reported that protein hydrolysates prepared using alkaline proteases have high antioxidant activity [49]. As previously stated, the CPE contained a higher concentration of hydrophobic amino acids, which have been reported in peptides with high antioxidant activity, than CP [50]. Polyphenols are one of the most important active components of Chlorella and have a wide variety of biological activities, including antioxidant activity, hyperlipidemia, and immune system regulation [51]. The TPC values of CP and CPE were 2.08 ± 0.12 and 29.06 ± 0.22 mM GAE/mL, respectively, showing that the TPC value of CPE was significantly higher than that of CP (*p* < 0.0001). In conclusion, CPE, the enzymatically hydrolyzed and extracted *C. pyrenoidosa* protein, had a significant amount of polyphenol compounds and a high degree of antioxidant activity, both of which have a beneficial influence on its potential as an alternative protein source.

## 4. Conclusions

This study focused on optimizing protein extraction from *C. pyrenoidosa* using microbial proteases. A quadratic equation was developed to predict an optimization model using variance-analyzed variables. Numerous factors were used in an RSM–BBD experiment to construct a numerical model to obtain equation coefficients for extracting *C. pyrenoidosa* proteins using microbial proteases. Single-factor analysis and RSM–BBD were used to identify the optimal microbial protease treatment factors for *C. pyrenoidosa* protein extraction. For optimum results, we used an enzymatic hydrolysis temperature of 45.5 °C, initial pH of 9.1, and hydrolysis time of 49.85 min. The optimization model was found to be statistically valid upon comparing the actual and anticipated values (*p* < 0.05). The total and free amino acid contents of CPE were higher than those of CP, and the essential amino acid content was also high. Moreover, CPE had a significant amount of polyphenols and antioxidant activity, making it a promising alternative protein source. The CPE optimized in this study is a low-molecular-weight protein that is simple to digest and absorb and might replace protein materials in protein food production. Further research is necessary to investigate the nutritional value of Chlorella proteins in animal models in vivo. Additional research is required to develop different methods for the application of proteins isolated from Chlorella. Additional research is also required to utilize the proteins extracted from Chlorella, such as the encapsulation of protein in a biocompatible polymeric material, as a safe dietary supplement.

## Figures and Tables

**Table 1:** Characteristics of enzymes and hydrolysis conditions for protein extraction from *Chlorella pyrenoidosa*.

Enzyme	Source	Enzyme Activity	Composition	Optimal Hydrolysis Conditions		Reference
				pH	Temperature (°C)	
Microbial protease	*Bacillus* sp.	≥16 U/g	Endopeptidase	10.0	50	[16]
Papain	*Carica papaya*	≥10 U/mg protein	Endopeptidase	6.0	50	[17]
Bromelain	Pineapple stem	≥3 U/mg protein	Endopeptidase	6.0	50	[18]
Cellulase	*Trichoderma reesei*	≥700 U/g	Cellulase	5.0	50	[19]
Viscozyme L	*Aspergillus* sp.	≥100 U/g	Hemicellulase	5.0	50	[20]

**Table 2:** Protein extraction yield from *Chlorella pyrenoidosa* upon treatment with enzymes.

Time (h)	Yield of Protein Extraction (%)					
	Microbial Protease	Papain	Bromelain	Cellulase	Viscozyme L	Control
0	10.52 ± 0.33 ^Ad^	7.11 ± 0.13 ^Be^	5.41 ± 0.23 ^Cd^	6.23 ± 0.25 ^BCa^	6.42 ± 0.11 ^BCd^	5.62 ± 0.12 ^Cd^
3	34.54 ± 2.48 ^Aa^	13.78 ± 1.29 ^Bd^	8.37 ± 0.86 ^Cc^	5.31 ± 0.55 ^Db^	7.76 ± 0.04 ^Cc^	5.51 ± 0.20 ^Dcd^
6	32.45 ± 2.73 ^Aa^	16.09 ± 0.29 ^Bbc^	9.21 ± 0.75 ^Cb^	6.57 ± 0.27 ^Da^	9.23 ± 0.70 ^Ca^	5.80 ± 0.11 ^Dcd^
9	32.72 ± 0.07 ^Aa^	15.47 ± 0.61 ^Bc^	9.58 ± 0.67 ^Cab^	4.80 ± 0.30 ^Ec^	7.55 ± 0.15 ^Dc^	5.89 ± 0.32 ^DEbc^
12	29.48 ± 2.05 ^Ab^	16.94 ± 0.80 ^Bab^	9.34 ± 0.44 ^Cab^	5.63 ± 0.08 ^Db^	8.40 ± 0.79 ^Ca^	6.21 ± 0.15 ^Db^
15	33.60 ± 3.16 ^Aa^	17.53 ± 0.51 ^Ba^	9.93 ± 0.24 ^Ca^	6.52 ± 0.47 ^Da^	8.68 ± 0.29 ^Cab^	6.65 ± 0.07 ^Da^
18	26.47 ± 0.65 ^Ac^	18.22 ± 0.47 ^Ba^	10.02 ± 0.32 ^Ca^	6.48 ± 0.24 ^Ea^	8.52 ± 0.30 ^Db^	6.80 ± 0.21 ^Ea^

Control, Chlorella suspension without enzyme treatment. Values with different letters vary significantly (*p* < 0.05). Uppercase letters compare the effect of each enzyme at the same hydrolysis time, and lowercase letters compare the effect of hydrolysis time for each enzyme.

**Table 3:** Box–Behnken experimental design of three variables and results of the yield of protein extraction from *Chlorella pyrenoidosa*.

No.	A, Hydrolysis Temperature (°C)	B, Initial pH	C, Hydrolysis Time (min)	Y, Yield of Protein Extraction (%)
1	40	9	40	35.07
2	40	11	40	31.74
3	50	9	30	36.59
4	50	10	40	37.88
5	60	10	50	36.41
6	40	10	30	32.25
7	40	10	50	36.91
8	60	11	40	33.50
9	50	10	40	37.88
10	60	9	40	35.75
11	50	10	40	37.88
12	50	11	50	38.02
13	60	10	30	35.95
14	50	9	50	39.39
15	50	10	40	37.88
16	50	11	30	38.16
17	50	10	40	36.45

**Table 4:** Analysis of variance and adequacy of the quadratic model.

Source	Sum of Squares	Degrees of Freedom	Mean Square	*F*-Value	*p*-Value
Model	65.19	9	7.24	7.58	0.0071
					Significant
A	3.99	1	3.99	4.18	0.0402
B	3.62	1	3.62	3.79	0.0426
C	7.57	1	7.57	7.92	0.0260
AB	0.29	1	0.29	0.31	0.5958
AC	4.43	1	4.43	4.63	0.0384
BC	2.16	1	2.16	2.26	0.1763
A^2^	39.23	1	39.23	41.06	0.0004
B^2^	0.64	1	0.64	0.67	0.4394
C^2^	4.00	1	4.00	4.19	0.0299
Residual	6.69	7	0.96		
Lack of Fit	5.02	3	1.67	4.01	0.1064
					Not significant
R^2^	0.9270				
Regression equation	Y = −99.86311 + 3.27244 A + 8.71967 B + 0.57823 C + 0.027155 AB − 0.010520 AC − 0.073515 BC − 0.030525 A^2^ − 0.39049 B^2^ + 0.00975255 C^2^				

Significant, *p* < 0.05; very significant, *p* < 0.01.

**Table 5:** Regression analysis for the quadratic model.

Source	Predicted Value (%)
Mean	39.96
Standard deviation	0.82
Model degree	Quadratic
R^2^	0.9270
Adequacy precision	10.749

**Table 6:** Total amino acid compositions of *Chlorella pyrenoidosa* powder (CP) and *C. pyrenoidosa* protein extracted by microbial protease (CPE).

Amino Acids	Concentration of Total Amino Acid (mg/100 g)		*t*-Value	*p*-Value
	CP	CPE		
Aspartic acid	3847.4 ± 26.4	5555.0 ± 90.3	−25.650	0.002
Threonine *	2036.0 ± 32.9	2934.0 ± 54.4	−18.601	0.003
Serine	1748.6 ± 35.1	2433.5 ± 85.1	−10.077	0.010
Glutamic acid	5032.5 ± 26.8	7104.2 ± 61.9	−79.657	0.000
Glycine	2539.1 ± 58.9	3353.6 ± 41.1	−52.056	0.000
Alanine	3763.1 ± 36.0	5591.8 ± 80.8	−42.933	0.001
Cystine	533.4 ± 13.7	760.9 ± 16.4	−67.131	0.000
Valine *	2813.2 ± 40.6	3524.0 ± 49.7	−42.430	0.001
Methionine *	945.9 ± 11.1	1363.4 ± 38.4	−21.709	0.002
Isoleucine *	1770.8 ± 44.3	2193.6 ± 30.5	−15.417	0.004
Leucine *	4161.3 ± 61.1	5223.7 ± 34.7	−54.192	0.000
Tyrosine	1586.0 ± 16.2	1932.3 ± 18.3	−41.479	0.001
Phenylalanine *	2473.6 ± 42.0	2927.1 ± 37.1	−10.223	0.009
Lysine *	3717.1 ± 22.5	3472.4 ± 17.4	10.996	0.008
Histidine *	923.4 ± 10.5	1210.9 ± 12.1	−313.688	0.000
Arginine	2801.0 ± 47.4	3474.2 ± 41.9	−16.966	0.003
Proline	2342.4 ± 43.4	3341.7 ± 55.7	−18.615	0.003
∑EAA	18,841.3 ± 129.2	22,849.1 ± 194.9	−103.755	0.000
∑NEAA	24,193.5 ± 80.6	33,547.2 ± 340.2	−41.255	0.001
TAA	43,034.8 ± 69.8	56,396.3 ± 517.7	−51.196	0.000

Abbreviations: EAA, essential amino acid; NEAA, non-essential amino acid; TAA, total amino acid. Significant, *p*-value < 0.05. * Essential amino acids.

**Table 7:** Free amino acid compositions of *Chlorella pyrenoidosa* powder (CP) and *C. pyrenoidosa* protein extracted by microbial protease (CPE).

Amino Acids	Concentration of Total Amino Acid (mg/100 g)		*t*-Value	*p*-Value
	CP	CPE		
Aspartic acid	13.6 ± 0.2	25.8 ± 0.6	−48.478	0.000
Threonine *	50.1 ± 1.3	96.0 ± 1.8	−58.641	0.000
Serine	17.3 ± 0.8	50.6 ± 1.3	−46.381	0.000
Glutamic acid	288.9 ± 7.9	541.4 ± 10.6	−71.645	0.000
Glycine	23.0 ± 2.7	61.8 ± 0.6	−30.563	0.001
Alanine	164.5 ± 6.9	357.4 ± 5.5	−118.647	0.000
Cystine	1.9 ± 0.5	183.2 ± 5.3	−60.848	0.000
Valine *	30.4 ± 1.0	102.4 ± 1.2	−59.216	0.000
Methionine *	8.1 ± 0.3	74.4 ± 2.8	−38.866	0.001
Isoleucine *	11.8 ± 0.5	183.6 ± 6.4	−43.774	0.001
Leucine *	17.0 ± 0.7	47.7 ± 0.7	−45.102	0.000
Tyrosine	6.9 ± 0.3	21.7 ± 0.8	−36.414	0.001
Phenylalanine *	22.9 ± 1.5	102.1 ± 1.8	−114.135	0.000
Lysine *	72.7 ± 2.3	139.3 ± 12.6	−10.943	0.008
Arginine	35.6 ± 1.4	68.7 ± 4.0	−14.871	0.004
Proline	121.3 ± 3.0	217.1 ± 7.0	−30.673	0.001
β-amino isobutyric acid	ND	650.8 ± 23.6	−47.725	0.000
γ-amino-n-butyric acid	9.9 ± 0.3	117.2 ± 5.5	−34.831	0.001
∑EAA	213.0 ± 4.4	745.5 ± 23.1	−40.604	0.001
∑NEAA	683.0 ± 20.3	2295.6 ± 54.2	−59.654	0.000
TAA	896.0 ± 24.7	3041.2 ± 74.7	−53.824	0.000

Abbreviations: EAA, essential amino acid; ND, not detected; NEAA, non-essential amino acid; TAA, total amino acid. Significant, *p*-value < 0.05. * Essential amino acids.

## Data Availability

Data is contained within the article and supplementary material.

## Supplementary Materials

The following supporting information can be downloaded at: https://www.mdpi.com/article/10.3390/foods13030366/s1, Table S1: Validation of the optimized model of enzymatic hydrolysis for chlorella protein extraction by microbial protease.
