# Supplementary material for: Optimization of Protease Treatment Conditions for Chlorella pyrenoidosa Protein Extraction and Investigation of Its Potential as an Alternative Protein Source
Source: Foods. 2024 Jan 23;13(3):366. doi: 10.3390/foods13030366 (PMC10855255; doi:10.3390/foods13030366)
Supplement: Supplementary file 1 [file foods-13-00366-s001.zip › foods-2820036-supplementary.pdf]

`

Supplementary Materials

Optimization of Protease Treatment Conditions for *Chlorella pyrenoidosa* Protein Extraction and Investigation of Its Potential as an Alternative Protein Source

Kyung-Jin Cho [1,2,3], Min-Ung Kim [1], Geum-Jae Jeong [1], Fazlurrahman Khan [2,3,4], Du-Min Jo [1,2,3] and Young-Mog Kim [1,2,3,*]

[1] Department of Food Science and Technology, Pukyong National University, Busan 48513, Republic of Korea
[2] Marine Integrated Biomedical Technology Center, The National Key Research Institutes in Universities, Pukyong National University, Busan 48513, Republic of Korea
[3] Research Center for Marine Integrated Bionics Technology, Pukyong National University, Busan 48513, Republic of Korea
[4] Institute of Fisheries Sciences, Pukyong National University, Busan 48513, Republic of Korea
* Correspondence: ymkim@pknu.ac.kr; Tel.: +82-51-629-5832

Table S1. Validation of the optimized model of enzymatic hydrolysis for chlorella protein extraction by microbial protease.

Dependent Variable	Actual Experiment variables	Predicted value (%)	Actual value (%)	Error (%)
Yield of protein extraction	Hydrolysis temperature 45.6°C<br>Initial pH 9.1<br>Hydrolysis time 49.85 min	39.96	40.70	1.27
